# 微流控纸芯片在环境分析检测中的应用

**DOI:** 10.3724/SP.J.1123.2020.09004

**Published:** 2021-08-08

**Authors:** Yu ZHANG, Ji QI, Feng LIU, Ning WANG, Xiyan SUN, Rong CUI, Jialuo YU, Jiaming YE, Ping LIU, Bowei LI, Lingxin CHEN

**Affiliations:** 1.烟台大学环境与材料工程学院, 山东 烟台 264003; 1. School of Environment and Materials Engineering, Yantai University, Yantai 264003, China; 2.中国科学院烟台海岸带研究所, 山东省海岸带环境过程重点实验室, 海岸带环境过程与生态修复重点实验室, 山东 烟台 264003; 2. Yantai Institute of Coastal Zone Research, Chinese Academy of Sciences, Shandong Provincial Key Laboratory of Coastal Environmental Process, Key Laboratory of Coastal Environment Processes and Ecological Remediation, Yantai 264003, China; 3.浙江清华长三角研究院分析测试中心, 国家食品安全风险评估中心应用技术合作中心, 浙江 嘉兴,314006; 3. Analysis and Testing Center of Zhejiang Tsinghua Yangtze River Delta Research Institute, Cooperation Center for Application Technology, China National Center for Food Safety Risk Assessment, Jiaxing 314006, China

**Keywords:** 微流控纸芯片, 芯片制造, 分析方法, 环境监测, 综述, microfluidic paper-based chips, chip fabrication, analytical methods, environmental monitoring, review

## Abstract

近年来,微流控纸芯片由于低成本、便携化、检测快等优点,在需要快速检测的环境分析领域中展现出了巨大的应用前景。该综述从微流控纸芯片在环境分析中的应用角度,总结归纳了微流控纸芯片在环境分析中的最新研究进展,并展望了其在未来的发展趋势与挑战。论文内容引用150余篇源于科学引文索引(SCI)与中文核心期刊中的相关论文。该综述包括微流控纸芯片在环境检测中的优势与制造方法介绍;电化学法、荧光法、比色法、表面增强拉曼法、集成传感法等基于纸芯片的先进分析方法介绍;根据环境分析目标物种类,如重金属离子、营养盐、农药、微生物、抗生素以及其他污染物等,对纸芯片的最新应用现状进行了举例评述;基于微流控纸芯片的环境分析研究的未来发展趋势和前景展望。通过综述近期相关研究,表明微流控纸芯片从提出至今虽然只有十几年的发展历程,但其在环境分析研究中的发展却十分迅速。微流控纸芯片可以根据不同的环境条件和检测要求灵活选择制作与分析方法,实现最佳的检测效果。但是微流控纸芯片也面临一些挑战,如纸张机械强度不足、流体控制程度不佳等问题。这些问题指出了微流控纸芯片在环境检测领域的发展趋势,相信随着不断深入的研究,纸芯片将会在未来的环境分析中发挥更大作用。

## Abstract (en)

Microfluidic paper-based chips have many advantages such as ease of integration, miniaturization, and automation; high throughput; low production cost; easy portability; easy storage and transportation, environmental friendliness, and feasibility of instantaneous detection. These chips are widely used in clinical diagnosis, food quality control, and immunoassays. With the continuous development of microfluidic paper microarrays in recent years, they have also received great attention for environmental contaminant analysis and detection, and research in this field has been intensive, showing excellent prospects for application. This review summarizes the latest research progress in environmental analysis from the perspective of the application of microfluidic paper-based chips, as well as future development trends and challenges. More than 150 papers from the Science Citation Index (SCI) and Chinese core journals are cited in this paper. This review includes the advantages of microfluidic paper-based chips for environmental analysis and detection; the introduction of paper chip fabrication methods, including wax printing, photolithography, dicing, plasma, laser, and inkjet etching; and the introduction of advanced analytical methods based on paper chips, such as electrochemical analysis, fluorescence analysis, colorimetric analysis, surface-enhanced Raman analysis, and integrated sensing methods. The future development trends and prospects of environmental analysis based on microfluidic paper-based chips are also reviewed. Through a rich and comprehensive review of recent related research, it is shown that although microfluidic paper-based chip technology has only been developed for little more than a decade since its introduction, this technology has seen rapid development in environmental analysis-related research and has yielded rich results. The hydrophilic and porous nature of cellulose in paper as a chip substrate allows the passive transport of liquids without an external power source. The diversity of available microfluidic paper-based chip fabrication and analysis methods allows flexible selection and matching according to different environmental conditions and detection requirements, so that the best detection results can be obtained. Moreover, microfluidic paper chips as detection platforms show good biocompatibility in the analysis and detection of environmental pollutants, enabling the analysis of more types of pollutants. The used paper is biodegradable and can be directly disposed of as ordinary garbage after appropriate degradation treatment; thus it is environmentally friendly and does not impact the health of the operators. In addition, the low production cost and simple operation of the paper chip design study make it possible to fabricate low-cost, portable, and practical analytical equipment, which is important for rapid testing of the conventional environment. However, there are some inherent disadvantages: the mechanical strength of the paper is not sufficiently high to resist deformation; degree of fluid control is difficult to achieve the desired effect, and the sample flow may be lost due to leakage; multiple contaminants may interfere with one another when analyzed in parallel; there are difficulties in commercial mass production. However, these problems also point to the direction for the research and development of microfluidic paper-based chips in the field of environmental testing. With continuous advances in manufacturing and analysis technologies, microfluidic paper-based chips are expected to play a more prominent role in future environmental analysis.

1990年,由Manz等^[1]^第一次提出微型全分析系统(miniaturized total analysis system, μTAS)这一名词后,微型分析平台的相关研究迅速发展。在这其中,微流控技术,又被称为芯片实验室、微流控芯片等,也在迅速崛起。它是对微观尺度流体能进行精准操作和控制的一类技术,其在微米尺度的芯片上,实现了分析处理全过程的高效化、微型化、集成化和便捷化操作^[2,3,4]^。微流控技术依靠运输便利性、样品及试剂量小、快速分析、能够进行平行处理等独特优势,在医疗领域、生物工程、环境监测等许多领域展现出了巨大的发展潜力。

微流控纸芯片(microfluidic paper-based chips)或微流控纸分析装置(microfluidic paper-based analytical devices, μPADs)是基于纸张加工微流体通道的微流控分析设备,同时也是微流控技术研究的前沿领域。微流控纸芯片与传统微流控芯片相比,具有独特优点:(1)纸张作为原材料,来源十分广泛。生产微流控芯片传统的制作材料石英、玻璃、硅片和有机聚合物等与纸相比,价格更高,其来源也不如纸张广泛。(2)运输方便,便携性高,可进行实时操作。纸张厚度小(0.06~1 mm),可折叠以方便保存和携带^[5]^。(3)无需外加动力源。流体可以通过纸张纤维素的毛细作用流动^[6]^,通过疏水化处理形成一定结构的通道可使流体有序流动。(4)生物兼容性优越。纸张的纤维素可被功能化,其亲水性、吸水性等可被改变^[7]^。(5)环境友好度高。纸芯片在使用后可通过点燃等简单方法进行处理,几乎不会对环境造成负担。

## 1 微流控纸芯片与环境监测

### 1.1 微流控纸芯片在环境检测分析中的意义

现代社会迅速发展带来的环境问题越来越多,对人们的身体健康和生活方式都造成了负面影响。工厂的违规排放、农药的滥用等对河流、湖泊、海洋等水体造成了严重污染。同样地,土壤环境与空气质量也在受到破坏,全球的生态平衡与生态安全在遭受着严重威胁。因此寻找一种低成本、易操作并且能够即时检测的方法进行环境检测显得尤为重要。纸芯片除了能够很好地执行常规环境监测外,还有其他独特优势:制作成本低,可以大量生产,增加监测的频率,使监测结果更加准确;高便携性和运输的便利性能够使监测的范围进一步扩大,使环境监测覆盖的范围更加广泛;其易上手和易操作可以让更多的人参与到环境监测中,同时也拓宽了监测结果的受众范围;即时检测能够为突发环境事件提供及时准确的反馈,能够使解决方法更加有针对性。因此,纸芯片在环境领域具有重要作用,是对环境进行监测分析的一类强大工具^[8,9]^。

### 1.2 纸芯片的制作方法

制作纸芯片,首先要选对纸张。能够制造纸芯片的纸张要有一定机械承受力,在液体中不会轻易变形;还要有良好的亲水或疏水性,以形成测试区域;另外化学性质要稳定,不与试样发生反应^[10]^。目前微流控纸芯片制作的基底材料通常选择吸水性能优异、不易变形且廉价易得的滤纸,其中表面光滑平整、性质均一、流体流速适中且颗粒留存度高的Whatman No.1滤纸是最常用的一种滤纸。此外,需要根据不同的检测要求选择不同类型的纸张^[11,12,13,14]^。

纸芯片制作的关键环节是通过构建亲疏水网络从而控制液体流动来制成二维(2D)纸芯片,还可通过叠加黏合或折纸法制成三维(3D)纸芯片^[15,16]^。依据构建亲疏水通道方法的不同可分为如下几类:通过疏水材料如石蜡、光刻胶、聚氨酯丙烯酸酯等在纸上构成疏水通道(对于有机溶剂不能采取此方法);使用聚合物如聚二甲硅氧烷(PDMS)、聚苯乙烯或聚亚胺酯对纸上孔洞进行堵塞形成物理屏障;通过物理切割直接形成通道。这些方法都有各自的优缺点,制作出的纸芯片也具有不同的特点,因此可以根据对芯片的不同需要选择不同的加工方法进行制作。

1.2.1 常见制作方法

蜡印法:蜡是用于制作μPADs最常见的材料,其成本低、易获得,化学性质相对不活泼。蜡印法只需将蜡块加热,使蜡融化渗入纸张,通过喷蜡打印机进行打印即可完成,而且通过印刷不同厚度或数量的蜡就能在纸上构造出半封闭和全封闭以及不同尺寸的通道。石蜡打印法是目前最常用的制作方法,可大批量生产对图案分辨率要求不高的纸芯片。

光刻法:光刻法是最早被用来创建疏水屏障的一类方法^[17]^。光刻胶能够使液体在纸上运动不泄漏,还能形成高分辨率图案,但光刻胶的低柔韧性导致制成的纸芯片易碎^[18]^。常用的负光胶SU-8,可以有效抵挡表面活性剂溶液和有机溶剂,但价格贵且工艺复杂。近期本实验室就采用聚氨酯丙烯酸酯(PUA)^[19]^这种光敏材料来制造低成本且环境友好的纸芯片,还有利用紫外光降解自组装硅烷化单分子层^[20]^以得到具有清晰图案且性质稳定的纸芯片。

切割法:切割技术是通过创建物理边界来构成通道或图案,使制造过程更简单,有工艺刀切割和激光切割两种方法。工艺刀切割是在电脑控制下进行制作,依据切割力度和角度的不同进行制作,可在底部加装保护层以避免纸张被刀割破;激光切割无需外加保护层但需要专门的激光切割仪器进行操作,与工艺刀相比操作难度增加,成本提高。Crooks等^[21]^用激光将设计好的通道切除,形成的中间镂空的夹心型纸芯片会让流体流动速度得到大幅度提升。

等离子体法:等离子体技术是利用等离子体发射设备以还原纸张的局部亲水性,因此经常出现在纸芯片亲水区域的制造环节中^[22,23]^。近期,Kao等^[24]^制造了一种电池供电的便携式微等离子体产生装置,可在一般条件下对疏水区进行化学改性,成为亲水区,摆脱了大型等离子仪器的桎梏,使等离子体装置的便携应用成为可能。

刻蚀法主要有激光刻蚀法、喷墨剂刻蚀法两种。激光刻蚀法利用激光对具有疏水层的纸张进行选择性改变,使其由疏水变为亲水^[25]^。与激光刻蚀法原理类似,喷墨剂刻蚀法利用的是喷墨剂对疏水纸张进行改性,针对不同物质形成的疏水层可以采用不同的喷墨剂进行刻蚀以形成亲水通道^[26,27]^。

一些纸芯片的制造方法示例见图1。

**图1:**
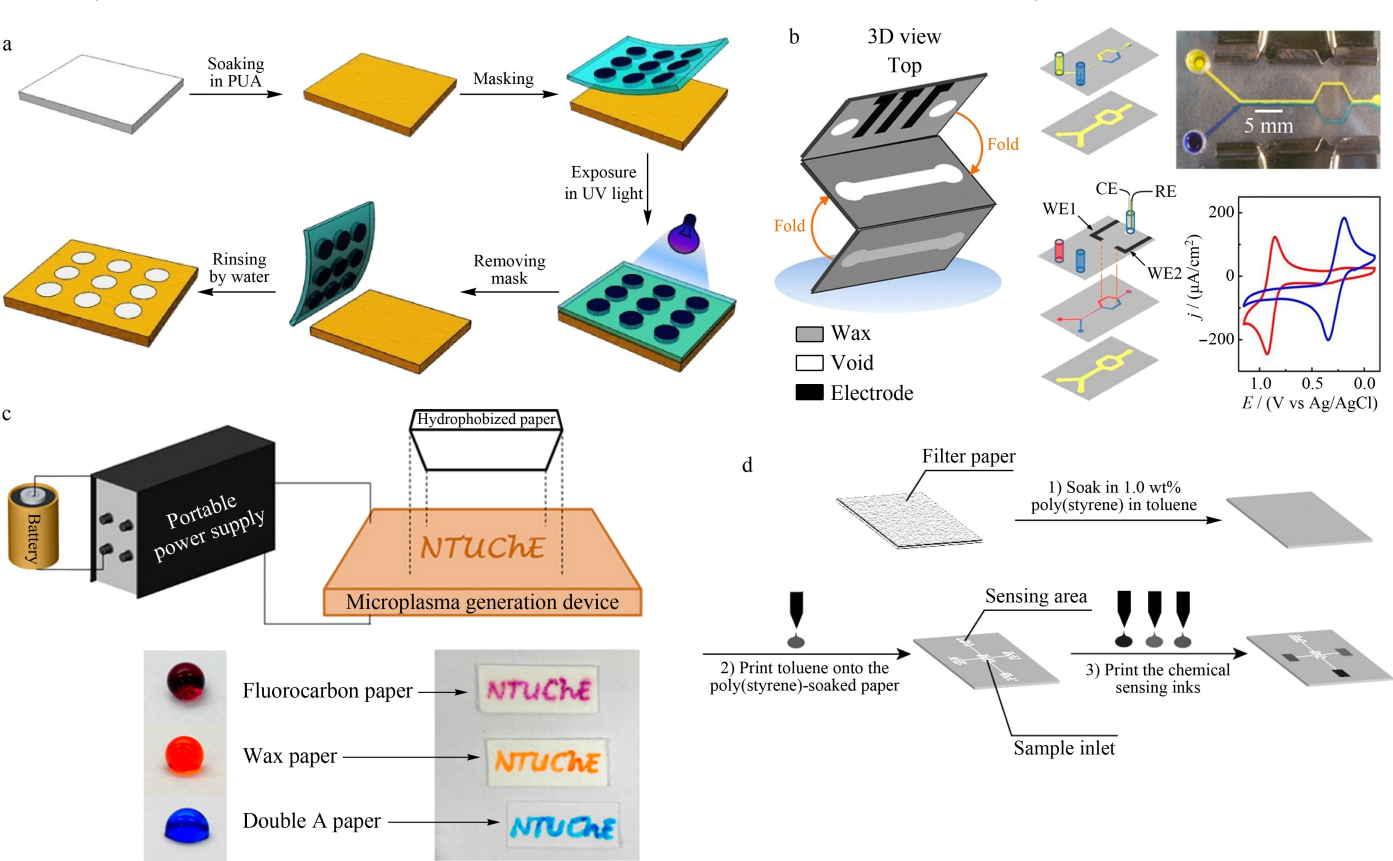
常见的纸芯片制作方法

1.2.2 其他方法

手工绘图简单方便,使用蜡笔等就可完成无需借助仪器,适合于制造图案简单、对分辨率要求不高且不接触有机溶剂的纸芯片。可以将印刷行业中常见的丝网印刷运用于纸芯片的制造,操作简单^[28]^,适合于大批量生产分辨率不高的纸芯片;融蜡浸透法^[29]^既无需借助仪器也不需要化学试剂,但制作出的纸芯片精度较低;柔印法^[30]^也是一种能快速生产大量纸芯片的方法,但由于要配备专用的柔性版印刷机和该打印机专用的单个印版,其设计灵活性受到了限制。

三维立体纸芯片:随着研究的深入和发展,除了传统的2D横向流纸芯片,能够实现纵横方向上均有通道、可使液体相互交叉不污染的3D纸芯片是一类应用前景广阔、发展潜力巨大的新型纸芯片,其出色的流体分配能力极大地拓展了分析能力,可以实现多目标的同时测定,分析效率得到大幅度提升。3D纸芯片的制作技术有用胶带或喷涂将单独的图案纸层黏合在一起的黏合法,以及将单层的图案纸简单折叠的折纸法^[31,32]^。

## 2 环境检测分析中微流控纸芯片的分析方法

环境检测分析中纸芯片的分析方法主要包括电化学法、发光法、比色法以及新兴的表面增强拉曼法(SERS)和集成移动感测平台法等。

### 2.1 电化学法

电化学法中的电极通常包括工作电极、对电极和参比电极3部分,具有高灵敏度和高选择性,其与纸芯片的兼容性极其优异。在纸芯片上制造电极的方法目前有使用石墨铅笔直接绘制电极^[33]^或用导电的碳或金属墨水丝网印刷或喷墨印刷电极^[34]^,也有研究采用掩模引导喷涂电极^[35]^。具有高导电率、高透明度、高机械强度以及巨大比表面积的石墨烯电极是电极材料的首选,通过涂抹氧化锌纳米颗粒^[36]^、铂纳米颗粒^[37]^等纳米级别的贵金属颗粒,可提高它和生物分子(DNA、蛋白质)的偶联能力,实现对生物分子更高水平的检测。电化学纸芯片可以通过折纸法形成3D结构^[38]^,为环境监测提供更好的分析平台,近期还有研究^[39]^将纸芯片与陶瓷或塑料电极组件进行结合,以提高使用次数和延长使用寿命。

### 2.2 光学分析法

2.2.1 发光法

发光法有荧光法、化学发光法、电化学发光法3种。荧光法选择性和灵敏度高,检出限低,但其需要借助仪器,也易受到纸张自身荧光剂的干扰;化学发光法和电化学发光法与荧光法相比更加灵敏^[40]^,无需激发源,所以背景光的信号更低,误差更小。但化学发光法只能通过试剂和待测物的物理混合来控制反应,而电化学发光法则可以通过控制在芯片上的激发时间和位置来实现更好的分析选择性^[41]^。

荧光法需要借助荧光仪进行分析,通常是检测待测样品表面结合的荧光团或纸上量子点的荧光猝灭^[42,43]^。据本课题组的现有研究表明^[42]^,水中重金属可通过荧光法在纸芯片上进行检测,现象明显,检测灵敏度高,测试效果良好。除重金属离子之外,也有研究团队开发出了其他污染物包括有毒化合物^[44]^、农药^[45]^、DNA^[46]^等的基于荧光法的纸芯片。

化学发光法的基本原理是通过化学反应在某个时刻的发射光强来分析某一组分的浓度。化学发光法所用的试剂大都价格低廉,但化学发光法的灵敏度很高,因此非常适合进行低成本的高灵敏度测定^[47,48,49,50]^。通过构建3D结构还可实现对多种样品进行平行检测^[51]^。

电化学发光法是由Delaney等^[52]^提出的一种将电化学技术与化学发光法相结合的新型方法,通过控制电位可以提高选择性,检测的动态浓度范围也有所扩大,并且兼容适用于化学发光法的所有试剂,是最受欢迎的分析方法之一^[53,54,55,56,57]^。

上述研究结果表明,基于发光法的纸芯片在自身优势的基础上,还可以与其他多种技术结合,不仅能够实现检测物种类上更大范围的检测,还在精度和灵敏度上也将会有相当程度的提高。而且发光法的现象明显,能够在结果的表达上进行简化,使检测过程更加简单。

2.2.2 比色法

比色法包括视觉、光度和反射检测3种方法^[58,59,60]^,通过显色反应,可以直接用肉眼观察颜色变化,进行半定量分析^[61]^,还可以采用相机拍照、扫描成像获得图片后利用图像软件(Photoshop、Image J等)进行定量分析,现在更倾向将其和智能设备集成,分析过程得到简化,分析效率提高^[62,63,64]^。比色法与其他方法如电化学法等相比,虽然检测结果会受到环境光线的影响,抗干扰性能有所不足,但是现象明显,有时无需借助专门的仪器通过肉眼就能进行初步分析,而且分析方法也相对简单,且易操作。随着智能设备如智能手机的不断发展,这些成像设备的发展已能够在一定程度上消除光线条件对成像的影响。因此,比色法是一类十分受欢迎且使用范围最广泛的纸芯片分析方法。

环境分析检测中最为典型的有关比色法的应用是对水中的硝酸盐、亚硝酸盐^[65,66]^和重金属离子^[67,68,69,70]^进行检测。基于比色法的纸芯片检测水中重金属离子是一种简单实用的强大工具,能够对铁、汞、铜、铬、铅等多种金属离子进行分析,操作简单方便,成本低廉,现象明显,且灵敏度高。

2.2.3 表面增强拉曼法

表面增强拉曼是一类分子振动光谱,增强分子的拉曼光谱信号,能够提高检测水平,从而实现更低的检出限。SERS能够进行极低浓度水平上的生化检测^[71]^,是生物医学和化学分析领域中的重要方法。基于SERS的纸芯片已广泛应用于水质分析^[72]^等领域,本课题组目前也正对基于SERS的纸芯片进行积极研究^[73]^,希望开发出更加灵敏、检出限更低的检测方法。Hariharan小组^[74]^设计的基于SERS的微流控纸芯片检测出了极低浓度的儿茶酚。这一研究表明,基于SERS的纸芯片可与和其他方法联用,拓展应用范围。

### 2.3 集成移动感测平台

随着现代科技的发展,将智能手机与纸芯片结合,构成新的集成移动感测平台,在更高水平上更好地发挥作用,成为一项备受关注的研究课题。将纸芯片与智能手机集成,可对空气颗粒物^[75]^、食品中的诺氟沙星^[76]^、水中的重金属^[77,78]^和农药^[79]^进行检测分析,而且核酸^[80]^等生物分子以及细菌^[81]^、病毒^[82]^等也能采用这种集成平台进行分析,简化了检测过程,降低了生产与检测成本。

本课题组^[83]^设计出一种依据光反射原理进行定量比色测定的便携式纸芯片设备,其体积小、重量轻,操作方便,并且自带读数功能,可以进行即时分析,是一类简单高效的检测分析工具。Chen等^[84]^设计出的双极电极电化学发光纸芯片,具有电池能量供应和智能手机读数功能。这些设计说明,智能手机与纸芯片结合的集成移动感测平台仍有许多方向值得深入研究,能够在环境监测领域中发挥更大的作用。

## 3 微流控纸芯片在环境检测分析中的应用

### 3.1 重金属离子检测

重金属广泛分布在生态环境中,如在大气圈、水圈、土壤圈^[85]^都可以找到重金属的踪迹。不仅难以对其进行生物降解,而且它能在生态系统中持续累积。即使在环境中含量极少的情况下也会对生物体造成危害,因此重金属污染一直是大众关注的环境问题。重金属种类繁多,不同的价态对环境造成的影响也有所区别,所以研发对重金属能够进行高灵敏度特异性检测和即时检测的方法,始终是重金属污染研究领域的重点关注方向。

目前已有许多基于比色法的μPADs能够对环境中的重金属离子进行检测分析。铜(Cu)作为日常生活和工业生产中常用的重金属,进入环境的方式多种多样。过量摄入Cu会对人体造成伤害,尤其会引起心脏和肾脏疾病^[86]^,目前采用比色法的纸芯片^[87,88]^可以检测到mg/L的Cu^2+^。铅(Pb)在环境中不可降解,相较于对成人的危害,儿童受到的伤害更大,会导致儿童生长发育缓慢^[89]^,近期有研究^[90]^采用比色法对Pb进行检测,可以检测到mg/L的Pb^2+^。汞(Hg)是剧毒有害金属,长期接触会对人类造成运动障碍,罹患冠心病的概率上升^[91]^,现有采用双层纸芯片结合未修饰的银纳米颗粒对其进行检测^[92]^,也有利用金纳米颗粒结合表面等离子体激元耦合的比色法,其检测灵敏度为50 nmol/L^[93]^。铬(Cr)也是一种工业生产中使用范围较为广泛的重金属材料,常见于金属表面处理、印染等行业中,可在水环境中长期存在并积累^[94]^。Cr在环境中的稳定态有Cr^3+^和Cr^6+^两种,目前也有基于比色法的纸芯片对河水^[95]^中的Cr^6+^进行检测。

除了最常采用的比色法,还可采用电化学方法,对上述重金属离子进行检测,能够检测到μg/L的重金属离子检出限的数量级可达^[96,97]^。目前本课题组^[98,99]^将荧光法的优势(灵敏度高、检出限低)与印迹技术的特长(选择性优异)相结合,实现了待测物的特异性检测。采用仅靠重力和毛细管力驱动的化学发光法^[100]^,对金属离子进行检测,能够检测到mg/L的金属离子,而且成本更低,操作简单。

在纸芯片上对多种重金属进行平行检测也是纸芯片领域的重点研究内容。平行检测在节省时间的同时降低了成本,分析效率也得到了显著提升。Mentele等^[101]^设计出一种包含4个检测区域的纸芯片,可以同时测量燃烧灰分中Fe、Cu和Ni 3种金属元素。Devadhasan等^[102]^提出一种利用3种生色试剂(茚三酮、石蕊和2-硝基苯甲酸试剂)能够同时检测Ni^2+^、Cr^6+^和Hg^2+^的纸质传感器,这种纸质传感器能够同时检测mg/L的Ni^2+^、Cr^6+^和 Hg^2+^,检测效率得到了明显提高。这表明平行检测的潜力十分巨大,值得进一步深入探索。

由于重金属离子种类繁多、价态多样,针对重金属离子检测的纸芯片种类也相应很多。图2展示了部分用于重金属离子检测的纸芯片。而且使用纸芯片检测样品中的重金属离子在灵敏度、选择性、检出限及线性范围等方面都展现出了良好的效果。同时,在研究开发能够对多种金属离子进行平行检测的纸芯片这一领域中,仍有极大的发展空间。以上这些例子均表明纸芯片在重金属离子检测领域具有很大的潜力,有进一步开发探索的价值。

**图2:**
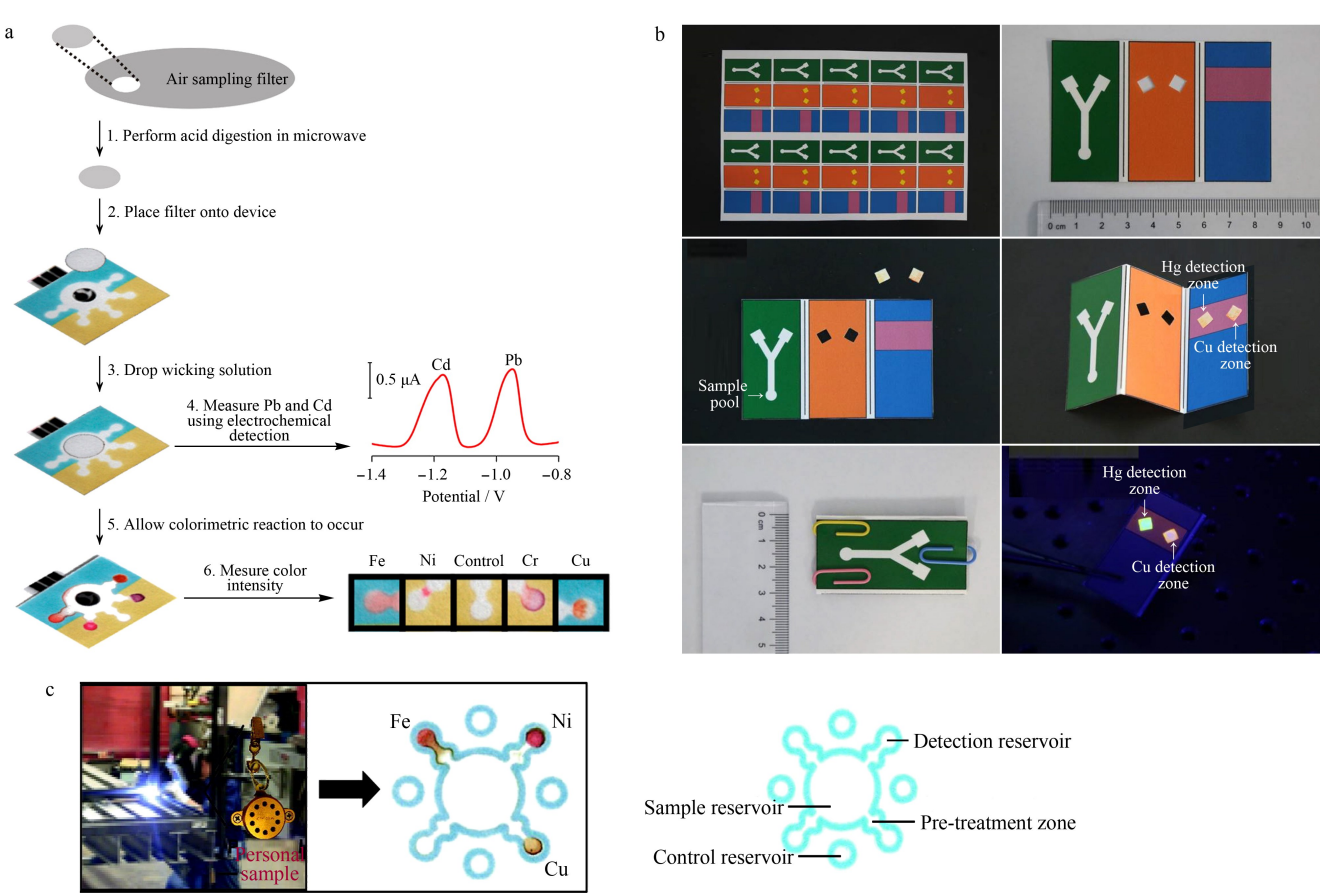
测定重金属离子的纸芯片

### 3.2 营养盐检测

大量无机盐尤其是硝酸盐、亚硝酸盐、磷酸盐等通过工业生产、畜牧养殖、农业灌溉进入到环境当中。含有氮磷化合物的废水进入水体,会造成水体富营养化,形成水华^[103]^;氮磷肥料的相关工业的扬尘排放、运输可能会对周围环境,尤其是土壤,造成环境污染,沉积的污染物则会对植物和地下水造成破坏^[104]^。

检测氨的纸芯片大多采用基于比色的气体扩散方法^[105,106]^。基于比色的检测装置包括两部分:含有氢氧化钠的圆形亲水层和酸碱指示剂区。指示剂区由膜(聚四氟乙烯胶带)或隔离物(没有膜)所形成的气体缝隙隔开。在待测物到达氢氧化钠层后,铵离子被转化为氨气,氨气通过疏水膜或气体缝隙进入指示剂区,导致比色剂发生变化。近期,还有课题组^[107]^发明出一种新型双层纸芯片用来测定淡水中的总氨,基本原理也是基于比色法的气体扩散。

亚硝酸盐作为常见的食品添加剂,容易和食物中的胺类物质发生反应产生致癌的亚硝胺化合物^[108]^,对人体健康造成危害。目前针对亚硝酸盐检测的纸芯片大多基于Griess反应(偶联反应)^[109,110]^,如图3a所示。硝酸盐和亚硝酸盐也是重要的水质参数,设计研发能够同时对亚硝酸盐和硝酸盐进行测定的装置也是研究的热点。最新的一种基于比色法的纸芯片不仅能同时测定硝酸盐、亚硝酸盐,还能测定硼砂和水杨酸^[111]^。此方法无需外接仪器,检测过程在相当程度上被简化,检测效率大幅度提高。

**图3:**
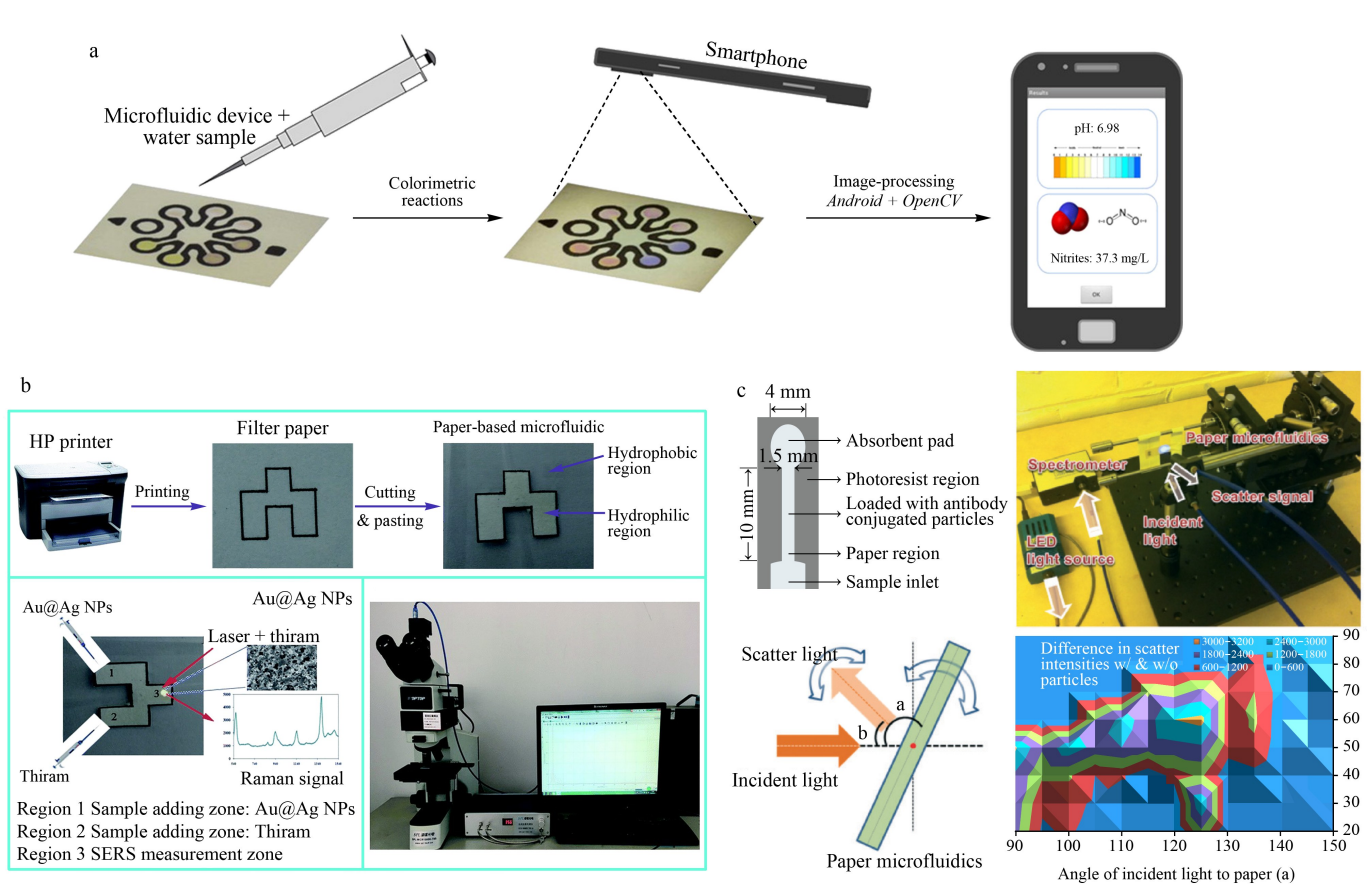
用于检测细菌、抗生素和多种污染物的纸芯片示例

环境中的磷酸盐也是近年来的重点研究对象。磷酸盐不仅会造成水华,还会对人体肾脏造成危害^[112]^,因此对磷酸盐进行快速灵敏的现场测定显得尤为重要。目前已有超灵敏检测海水中磷酸盐的比色法纸芯片^[113]^,也有基于荧光法与智能手机集成的微流控纸芯片检测环境中的磷酸盐^[114]^。针对土壤中的磷酸盐,目前也有一种新型低成本微流控纸质分析仪^[115]^可以对其进行检测,在此分析设备上最多可进行15次活性磷酸盐的重复测定,大大降低了检测成本,设备利用率得到显著提高。

近些年来,人们备受环境中营养物质污染带来的困扰,因此对这些营养物质进行常规定期监测十分必要。检测氨和亚硝酸盐的传统方法是采用分光光度法进行检测,而磷酸盐也通常采用电化学方法等。这些传统方法虽然灵敏度较高,但都依赖于大型仪器,局限于实验室之中,无法进行现场检测。然而,纸芯片这种成本低、易操作的分析测试方法能够很好地执行这种常规检测,并且能够进行现场即时检测,解决了传统检测方法存在的问题。还可以在纸芯片上针对不同的样品设计不同的预处理区域,这与传统方法相比节约了测试时间,测试效率得到提升。同时在灵敏度、检出限、特异性等方面与传统方法相比,差异并不明显。通过上述研究能够看出纸芯片在营养物污染这一领域有很大的发展空间,通过结合更多、更新的方法,能够实现更多种类、更大范围的营养物污染监测。

### 3.3 农药检测

农药在保护植物和农作物不受病虫侵害^[116]^的同时,其滥用也对环境造成了严重污染,河流、土壤、空气甚至日常的食物中都能找到农药的踪迹。液相色谱^[117,118]^、液相色谱-质谱(LC-MS)^[119]^等方法是测定农药的常用技术,但这些方法大都需要借助仪器,成本高,操作难度大,测试时间长,不适于现场测定。纸芯片是一种行之有效的解决方案,无需借助大型仪器,能够进行快速现场测定,更重要的是对测试人员的要求不高,生产成本也更低,对经济不发达地区农药污染的监测与治理意义重大。目前已开发出多种测定环境中农药的纸芯片,如Zhu等^[120]^将SERS结合微流控纸芯片对福美双(thiram)进行高灵敏度检测(见图3b);Hossain等^[121]^则开发出监测环境中有机磷酸酯农药的纸芯片。相信随着更加深入的研究,纸芯片将能够对更多种类的农药进行分析测定,为农药污染治理提供更有力的工具。

农药为农业生产带来便利的同时,也为生态环境带来了极大挑战。农药污染会直接危害到人类的身体健康,需要对其进行长期的常规检测,而传统的检测方法超高效液相色谱、液相色谱-质谱、酶联免疫吸附试验等无法对农药进行简单灵活、经济廉价的检测。但纸芯片却能够摆脱传统方法对大型仪器的依赖,能够进行成本低廉的即时检测和多次检测,同时纸芯片的检测结果也表现出了良好的灵敏度、重复性与稳定性,为农药污染的监测方法带来了潜在可能和新的发展方向。

### 3.4 微生物检测

微生物广泛分布在生态环境中,对生态系统和生物健康都有着重大意义,因此开发一种简单方便、价格低廉的微生物检测方法向来是环境监测领域的重要研究方向。传统检测方法是在实验室中对样品进行培养分析或通过聚合酶链反应、酶联免疫吸附测定等方法进行分子分析。这些方法测试时间长,操作条件复杂,对操作人员要求高。与之相比,纸芯片成本低,操作简单,能很好地完成常规测试。

目前应用μPADs进行检测的细菌主要有大肠杆菌、沙门氏菌和单核细胞增生性李斯特菌,可以检测沙门氏菌的纸芯片如图3c所示^[81]^。致病性大肠杆菌可通过饮水、食物等途径引起腹泻等多种疾病,严重者会有生命危险,现已开发出多种μPADs检测大肠杆菌的方法^[122,123,124]^;沙门氏菌与许多食源性疾病有关,可引起多种肠胃疾病,目前已有基于比色法的纸芯片^[125]^和集成智能手机和纸芯片的分析装置等对其进行检测;单核细胞增生性李斯特菌也是一种常见的病原体,它引起的李斯特菌病虽然流行程度不高,但相比之下死亡率却很高(20%~30%)^[126]^,现有一种RNA标记测定方法是在基于金纳米颗粒的纸质平台上进行的,整个分析过程可在几小时内完成^[127]^。还有一种对农业用水中大肠杆菌、沙门氏菌和单核增生李斯特菌浓缩、富集和检测集于一体的基于比色法的μPAD^[128]^。

微生物本身是自然界的一部分,但如果数量失去控制或者出现在本不应出现的环境中,同样会导致环境问题。与农药污染的传统检测方法类似,对于微生物的检测方法以往也是在实验仪器的桎梏之中,采用电化学法、光学方法完成核酸检测特定基因目标,这些方法都需要特定的设备和培训。简单高效的纸芯片则无需培训易于上手,体积微小具有运输上的便利性,更易于进行现场化检测,这对检测不同地区、不同环境中的微生物具有积极意义。目前纸芯片检测微生物的水平与传统方法相比尚有一定距离,但其为环境微生物的检测提供了一种新的思路,可以在此基础之上继续优化,以在未来的应用中发挥更大的作用。

### 3.5 新兴污染物抗生素的检测

随着现代科技的发展,环境中污染物的种类与数量也在与日俱增。这其中,就包括在医疗领域广为使用的抗生素。抗生素自被发现和使用后,在医学方面为人类带来巨大福祉的同时,其滥用对环境造成的污染也一直是环境研究中的重点问题。抗生素通常在畜牧业和水产养殖行业中^[129]^,作为促进生长或用来预防细菌感染^[130]^的药剂添加在动物的饲料和饮水中。这种使用方式极大地促进了细菌耐药性的增长,而且过度使用,会造成基因污染,很有可能催生出“超级细菌”。因此,对环境中的抗生素进行检测意义重大。传统方法通常采用LC-MS/MS^[131]^对抗生素进行检测,此方法需借助大型仪器,对测试人员的操作要求高,需要大量样品进行净化、浓缩^[132]^。近期,Nilghaz等^[133]^研究出一种基于微流控纸芯片的比色法检测猪肉中土霉素和诺氟沙星残留物,克服了传统方法的固有短板,为抗生素的检测方法提供了新思路。

能够对多种污染物同时进行检测的微流控纸芯片向来是纸芯片研究领域的重点方向。近期,Xing等^[134]^就制作出一种纸芯片可以同时检测饮用水中5种化学物质,利用基于抗体-抗原反应的多组分侧向流动检测技术同时检测铅、微囊藻毒素,氯霉素,睾丸激素和百菌清,整个检测过程仅需20 min即可完成,高效便捷。

关于抗生素这类新兴污染物的检测方法,发展初期大部分还是采用大型仪器联用,实现了高精度检测的同时也带来了高昂的成本,这其中既有仪器的费用也包括不菲的人工成本。运用纸芯片对抗生素检测,则无需考虑上述问题。

### 3.6 其他污染物检测

许多炸药的成分如2,4,6-三硝基甲苯(TNT)都具有高毒性,而且可在环境中长期存在,不合理的处置方式会使炸药对空气、土壤、水体产生污染^[135,136,137]^,对环境造成严重危害。目前用于炸药检测的方法有很多,如荧光法^[138]^、LC-MS^[139]^、CE-MS^[140]^等。这些方法在具有高灵敏度和特异选择性等优点的同时也存在成本高、操作难度大、便携性差等缺陷,使得其在实际应用中无法完全发挥作用。而纸芯片的高度便携性可以对爆炸前后进行即时检测,快速得到结果,不仅对环境检测具有重要作用,对机场、火车站等场所的公共安全也具有重要意义。目前已开发出多种基于化学发光法^[141]^、SERS法、^[142]^、荧光法^[143,144]^、比色法^[145]^的微流控纸芯片,用以检测环境中污染物,图4a就展示了一种检测爆炸物的纸芯片。Pesenti等^[146]^设计出的基于比色法的纸芯片,可同时检测3种三硝基芳族炸药TNT、三硝基苯(TNB)和三硝基苯甲硝胺(Tetryl)。

**图4:**
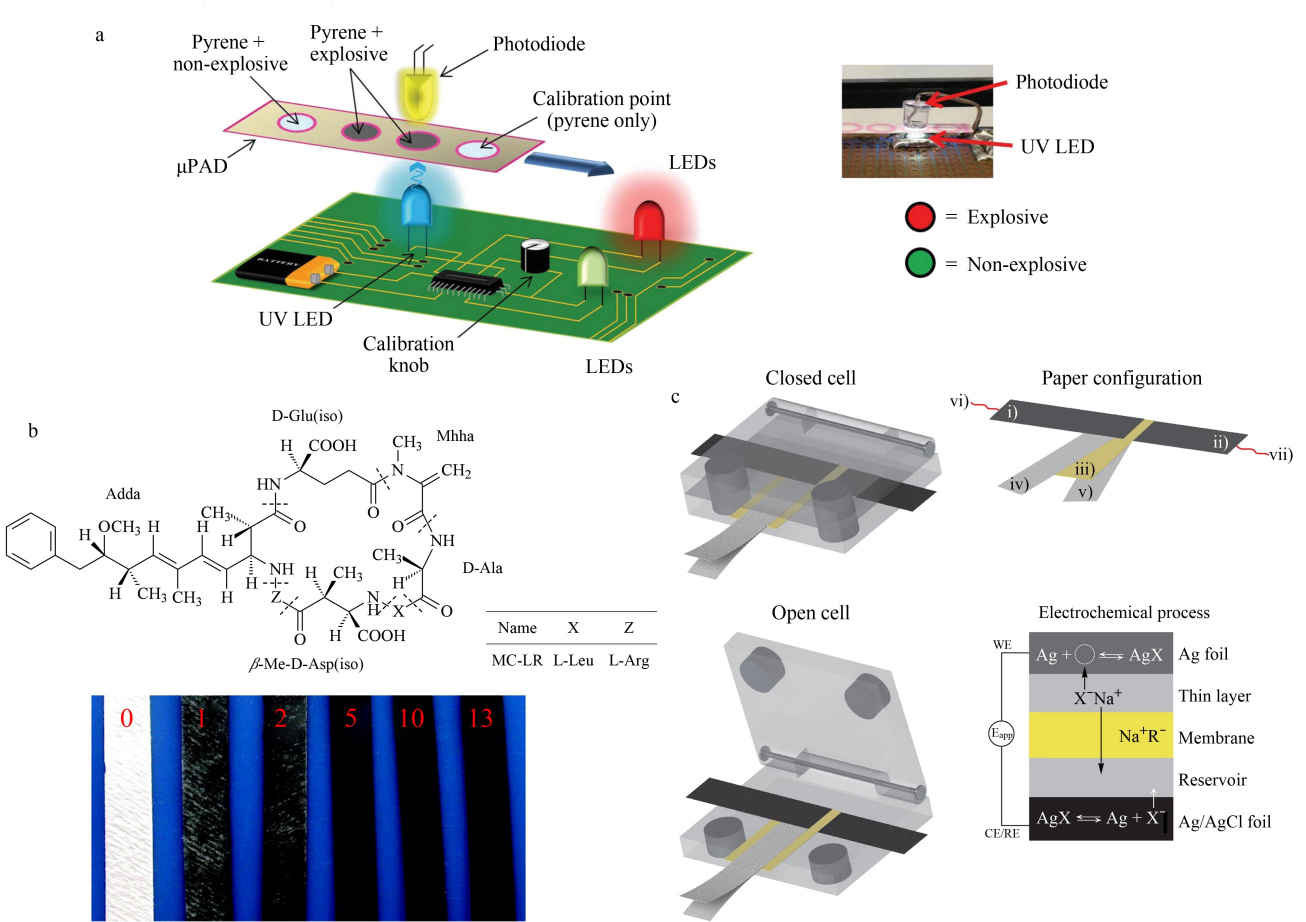
用于检测炸药、藻毒素和卤化物的纸芯片示例

藻毒素是蓝藻在淡水生态系统中产生的高毒性次生代谢产物^[147]^,藻毒素中毒性最高的是微囊藻毒素-LR(MC-LR)^[148]^,会造成严重的水质污染,对生物体造成危害^[149]^。检测水中MC-LR的方法很多,但要找出一种既简单方便又快速廉价的检测手段仍是一项重要课题。Ge等^[150]^利用金纸工作电极(Au-PWE)专门对水中的MC-LR进行电化学免疫测定;Wang等^[151]^开发出一种采用三电极的基于碳纳米管的纸芯片测定MC-LR,如图4b所示,其检测效果可与酶联免疫吸附测定相媲美。

还有一些污染物,比如碘^[152]^、溴^[153]^、氯^[154]^等卤族元素,以及溴化物^[153]^和其他卤化物^[152]^,目前也开发出相应的纸质传感器,能够对上述物质进行即时、高灵敏度的现场测定(见图4c)。相对于以往采用的检测方法,优势明显,是一种简单实用的测定工具。

微流控纸芯片良好的兼容性使得其能够对环境中的多种污染物进行检测,这在相当大的程度上扩展了纸芯片在环境监测领域中的应用范围。而且纸芯片与其他技术联用,表现出更加优异的性能,表明了纸芯片具有巨大的创新性与发展潜力。同时,适当的样品预处理方式也会对纸芯片检测环境样品的过程产生积极作用。环境中的污染物会以多种形式存在,这就造成了测试样品的繁杂种类,不同形态的样品都有可能出现在测试过程中,针对不同样品进行不同的预处理会使检测过程更加高效便捷。对于成分比较复杂的样品,分离与提纯是必要的预处理过程,可以在纸芯片外完成也可以在纸芯片上进行。纸芯片自身的材质使得其具有一定过滤作用,可以设计通道使样品在通往测试区域时就已完成过滤,也可以在纸芯片上利用纸张纤维素的毛细作用设计专门区域进行分离提纯区域,减少预处理时间。检测浓度极低的样品则可以在纸芯片上开发出富集与浓缩区域,以提高检测的灵敏度。若进一步将这些预处理区域与测试区域集成,则测试所需时间相应缩短,整体的检测效率随之提升。相信随着制作方法与分析技术的提高,以及预处理方法的合理选择,纸芯片的设计将得到不断优化与发展,呈现出更加卓越的效果和更加强大的功能。

## 4 结论与展望

虽然微流控纸芯片最早是为生物医学检测领域服务,但其经济、高效、耗样量少、对环境友好、易制造和操作、能迅速进行检测等多种优势使得在环境检测分析中的应用也日渐广泛。同时它作为多学科交叉的新兴技术,可以和许多现代技术如生物工程、纳米材料、数码科技等相结合,制作方法和流程也在与时俱进。而且与其在生物医学领域已经取得的成就相比,纸芯片在环境监测方面仍有广阔的发展空间。

因此,纸芯片的制造和分析技术仍需要改进,去迎接未来发展的挑战,如机械强度不高易变形破碎,流体的操控程度不够高,多种样品平行检测技术仍不够成熟等,这些缺陷的克服都需要进一步提高测试灵敏度和现场快速测试的能力,从而增加微流控纸芯片的实际应用的价值,使其能够在未来污染物检测分析领域中担任更加重要的角色。对微流控纸芯片的改进具体可从以下几方面进行:增加纸芯片的机械强度,使其更加坚韧不易变形;继续减少样品和试剂的消耗量,将检测成本降到更低;开发更多的检测分析方法与纸芯片相结合,实现多重分析;加强对流体的操控,使流体更加有序可控;降低多重分析中不同流体之间的相互干扰;扩大检测种类和范围;对纸芯片结构进行改造,实现多技术多分析物检测;针对不同类型的样品选择适当的预处理方法,设计不同的预处理区域,使检测过程更加高效便捷;开发更廉价的生产方法,实现纸芯片的产业化应用等。相信随着微流控相关研究与技术的发展,微流控纸芯片将会在未来环境污染物的检测分析中起到更大作用。

## References

[1] Manz A, Graber N, Widmer H M. Sensor Actuat B: Chem, 1990,1:244

[2] Li H F, Zhang Q Y, Lin J M. Chinese Journal of Chromatography, 2019,29(4):284 10.3724/sp.j.1123.2011.00284 21770235

[3] Lin B C, Qin J H. Chinese Journal of Chromatography, 2005,23(5):456 16350786

[4] Qin J H. Chinese Journal of Chromatography, 2010,28(11):1009 21381413

[5] Jiang Y, Ma C C, Hu X Q, et al. Progress in Chemistry, 2013,26(1):167

[6] Tian T, Huang Y S, Lin B Q, et al. Journal of Instrumental Analysis, 2015,34(3):257

[7] Liana D D, Raguse B, Gooding J J, et al. Sensors (Basel), 2012,12(9):11505 23112667 10.3390/s120911505 PMC3478794

[8] Zang D J, Ge L, Yan M, et al. Chem Comm, 2012,48(39):4683 22301713 10.1039/c2cc16958d

[9] Qi J, Li B W, Zhou N, Wang X Y, et al. Biosens Bioelectron, 2019,142:11533 10.1016/j.bios.2019.111533 31377573

[10] Lee J W, Lee D, Kim Y T, et al. Biosens Bioelectron, 2017,91:388 28061421 10.1016/j.bios.2016.12.053

[11] Lei K F, Andrew G, Huang C H. Talanta, 2019,205:120124 31450396 10.1016/j.talanta.2019.120124

[12] Cate D M, Adkins J A, Mettakoonpitak J, et al. Anal Chem, 2015,87(1):19 25375292 10.1021/ac503968p

[13] Batule B S, Seok Y, Kim M G. Biosens Bioelectron, 2020,151:112400 10.1016/j.bios.2020.112400 32729520

[14] Al-Tamimi M, Shen W, Zeineddine R, et al. Anal Chem, 2012,84(3):1661 22148716 10.1021/ac202948t

[15] Yu L J, He Z W, Wei M Q, et al. Chinese Journal of Analysis Laboratory, 2016,35(5):611

[16] Chaiyo S, Apiluk A, Siangproh W, et al. Sensor Actuat B: Chem, 2016,233:540

[17] Martinez A W, Phillips S T, Butte M J, et al. Angew Chem Int Ed, 2007,46(8):1318 10.1002/anie.200603817 PMC3804133 17211899

[18] Dungchai W, Chailapakul O, Henry C S. Analyst, 2011,136(1):77 20871884 10.1039/c0an00406e

[19] Lin D, Li B W, Qi J, et al. Sensor Actuat B: Chem, 2020,303:127213

[20] He Q H, Ma C C, Hu X Q, et al. Anal Chem, 2013,85(3):1327 23244032 10.1021/ac303138x

[21] Renault C, Anderson M J, Crooks R M. J Am Chem Soc, 2014,136(12):4616 24635569 10.1021/ja4118544

[22] Li X, Tian J F, Nguyen T, et al. Anal Chem, 2008,80(23):9131 19551982 10.1021/ac801729t

[23] Yan C F, Yu S Y, Jiang Y, et al. Acta Chimica Sinica, 2014,72(10):1099

[24] Kao P K, Hsu C C. Anal Chem, 2014,86(17):8757 25052546 10.1021/ac501945q

[25] Chitnis G, Ding Z W, Chang C L, et al. Lab Chip, 2011,11(6):1161 21264372 10.1039/c0lc00512f

[26] Abe K, Suzuki K, Citterio D. Anal Chem, 2008,80(18):6928 18698798 10.1021/ac800604v

[27] Wang J Y, Monton M R N, Zhang X, et al. Lab Chip, 2014,14(4):691 24352569 10.1039/c3lc51313k

[28] Ma S, Tang Y Y, Liu J Q, et al. Talanta, 2014,120:135 24468352 10.1016/j.talanta.2013.12.007

[29] Songjaroen T, Dungchai W, Chailapakul O, et al. Lab Chip, 2012,12(18):3392 22782449 10.1039/c2lc21299d

[30] Olkkonen J, Lehtinen K, Erho T. Anal Chem, 2010,82(24):10246 21090744 10.1021/ac1027066

[31] Liu H, Xiang Y, Lu Y, et al. Angew Chem Int Ed, 2012,51(28):6925 10.1002/anie.201202929 PMC3486962 22639438

[32] Noiphung J, Songjaroen T, Dungchai W, et al. Anal Chim Acta, 2013,788:39 23845479 10.1016/j.aca.2013.06.021

[33] Li W B, Qian D P, Wang Q H, et al. Sensor Actuat B: Chem, 2016,231:230

[34] Ge L, Wang S W, Ge S G, et al. Chem Commun (Camb), 2014,50(43):5699 24904944 10.1039/c3cc49770d

[35] Santhiago M, Henry C S, Kubota L T. Electrochim Acta, 2014,130:771

[36] Sun G Q, Zhang L N, Zhang Y, et al. Biosens Bioelectron, 2015,71:30 25884731 10.1016/j.bios.2015.04.007

[37] Sun Y M, He K, Zhang Z F, et al. Biosens Bioelectron, 2015,68:358 25603401 10.1016/j.bios.2015.01.017

[38] Hasanzadeh M, Shadjou N. Mater Sci Eng C, 2016,61:979 10.1016/j.msec.2015.12.031 26838927

[39] Kong F Y, Gu S X, Li W W, et al. Biosens Bioelectron, 2014,56:77 24469540 10.1016/j.bios.2013.12.067

[40] Ge L, Yu J H, Ge S G, et al. Anal Bioanal Chem, 2014,406(23):5613 24705955 10.1007/s00216-014-7756-1

[41] Almeida M I G S, Jayawardane B M, Kolev S D, et al. Talanta, 2018,177:176 29108573 10.1016/j.talanta.2017.08.072

[42] Wang X R, Li B W, You H Y, et al. Chinese Journal of Analytical Chemistry, 2015,43(10):1499

[43] Chen X C, Yu S M, Yang L, et al. Nanoscale, 2016,8(28):13669 27376510 10.1039/c6nr02878k

[44] Petruci J F D, Cardoso A A. Anal Chem, 2016,88(23):11714 27807968 10.1021/acs.analchem.6b03325

[45] Ma X, Hao G Y, Zhang Z, et al. Scientia Sinica Chimica, 2020,50(3):393

[46] Scida K, Li B, Ellington A D, et al. Anal Chem, 2013,85(20):9713 24070108 10.1021/ac402118a PMC3852662

[47] Yu J H, Ge L, Huang J D, et al. Lab Chip, 2011,11(7):1286 21243159 10.1039/c0lc00524j

[48] Li W P, Ge S G, Wang S M, et al. Luminescence, 2013,28(4):496 23355319 10.1002/bio.2482

[49] Wang Y H, Wang S M, Ge S G, et al. Monatshefte Fur Chemie, 2014,145(1):129

[50] Liu W, Guo Y M, Li H F, et al. Spectrochim Acta A Mol Biomol Spectrosc, 2015,137:1298 25306129 10.1016/j.saa.2014.09.059

[51] Li F, Liu J C, Guo L, et al. Biosens Bioelectron, 2019,141 10.1016/j.bios.2019.111472 31272061

[52] Delaney J L, Hogan C F, Tian J, et al. Anal Chem, 2011,83(4):1300 21247195 10.1021/ac102392t

[53] Liu R, Zhang C S, Liu M. Sensor Actuat B: Chem, 2015,216:255

[54] Yan J X, Ge L, Song X R, et al. Chem Eur J, 2012,18(16):4938 22392821 10.1002/chem.201102855

[55] Ge L, Yan J X, Song X R, et al. Biomaterials, 2012,33(4):1024 22074665 10.1016/j.biomaterials.2011.10.065

[56] Yan J X, Yan M, Ge L, et al. Chem Commun (Camb), 2013,49(14):1383 23187464 10.1039/c2cc37402a

[57] Doeven E H, Barbante G J, Kerr E, et al. Anal Chem, 2014,86(5):2727 24512565 10.1021/ac404135f

[58] Klasner S A, Price A K, Hoeman K W, et al. Anal Bioanal Chem, 2010,397(5):1821 20425107 10.1007/s00216-010-3718-4

[59] Jayawardane B M, McKelvie I D, Kolev S D. Anal Chem, 2015,87(9):4621 25855368 10.1021/acs.analchem.5b00125

[60] Phansi P, Sumantakul S, Wongpakdee T, et al. Anal Chem, 2016,88(17):8749 27464645 10.1021/acs.analchem.6b02103

[61] Mujawar L H, Felemban A A, Shahawi M S. Aanl Sci, 2016,32(5):491 10.2116/analsci.32.491 27169646

[62] Cardoso T M G, Garcia P T, Coltro W K T. Anal Methods, 2015,7(17):7311

[63] Yetisen A K, Martinez-Hurtado J L, Garcia-Melendrez A, et al. Sensor Actuat B: Chem, 2014,196:156

[64] Fronczek C F, Park T S, Harshman D K, et al. RSC Advances, 2014,4(22):11103

[65] Jayawardane B M, Wei S, McKelvie I D, , et al. Anal Chem, 2014,86(15):7274 25001619 10.1021/ac5013249

[66] Ortiz-Gomez I, Ortega-Munoz M, Salinas-Castillo A, et al. Talanta, 2016,160:721 27591668 10.1016/j.talanta.2016.08.021

[67] Ogawa K, Kaneta T. Anal Sci, 2016,32(1):31 26753702 10.2116/analsci.32.31

[68] Apilux A, Siangproh W, Praphairaksit N, et al. Talanta, 2012,97:388 22841097 10.1016/j.talanta.2012.04.050

[69] Ratnarathorn N, Chailapakul O, Henry C S, et al. Talanta, 2012,99:552 22967593 10.1016/j.talanta.2012.06.033

[70] Li M S, Cao R, Nilghaz A, et al. Anal Chem, 2015,87(5):2555 25645265 10.1021/acs.analchem.5b00040

[71] Jia M, Li S M, Zang L G, et al. Nanomaterials (Basel), 2018,8(9):664 10.3390/nano8090730 PMC6165412 30223597

[72] Lee J C, Kim W, Choi S. Int J Pr Eng Man-GT, 2017,4(2):221

[73] Li B W, Chen L X. Journal of Instrumental Analysis, 2015,34(3):302

[74] Hariharan A, Chelli S M, Muthukumar V S, et al. Opt Mater, 2019,94:305

[75] Sun H, Jia Y, Dong H, et al. Anal Chim Acta, 2018,1044:110 30442391 10.1016/j.aca.2018.07.053

[76] Trofimchuk E, Nilghaz A, Sun S, et al. J Food Sci, 2020,85(3):736 32017096 10.1111/1750-3841.15039

[77] Nemiroski A, Christodouleas D C, Hennek J W, et al. Proc Natl Acad Sci U S A, 2014,111(33):11984 25092346 10.1073/pnas.1405679111 PMC4143067

[78] Liu X, Liu C, Li N, et al. Chinese Journal of Analysis Laboratory, 2017,36(1):120

[79] Sicard C, Glen C, Aubie B, et al. Water Res, 2015,70:360 25546358 10.1016/j.watres.2014.12.005

[80] Priye A, Wong S S, Bi Y P, et al. Anal Chem, 2016,88(9):4651 26898247 10.1021/acs.analchem.5b04153 PMC4857158

[81] Park T S, Li W Y, McCracken K E, , et al. Lab Chip, 2013,13(24):4832 24162816 10.1039/c3lc50976a

[82] Magro L, Jacquelin B, Escadafal C, et al. Sci Rep, 2017,7:1347 28465576 10.1038/s41598-017-00758-9 PMC5431003

[83] Li B W, Fu L W, Zhang W, et al. Electrophoresis, 2014,35(8):1152 24375226 10.1002/elps.201300583

[84] Chen L, Zhang C S, Xing D. Sensor Actuat B: Chem, 2016,237:308

[85] Liu X M, Song Q J, Tang Y, et al. Sci Total Environ, 2013,463/464:530 10.1016/j.scitotenv.2013.06.064 23831799

[86] Reddy S A, Reddy K J, Narayana S L, et al. Food Chem, 2008,109(3):654

[87] Chaiyo S, Siangproh W, Apilux A, et al. Anal Chim Acta, 2015,866:75 25732695 10.1016/j.aca.2015.01.042

[88] Jayawardane B M, Coo L, Cattrall R W, et al. Anal Chim Acta, 2013,803:106 24216203 10.1016/j.aca.2013.07.029

[89] Mahaffey K R. Pediatrics, 1977,59(3):448 840565

[90] Satarpai T, Shiowatana J, Siripinyanond A. Talanta, 2016,154:504 27154707 10.1016/j.talanta.2016.04.017

[91] Counter S A, Buchanan L H. Toxicol Appl Pharmacol, 2004,198(2):209 15236954 10.1016/j.taap.2003.11.032

[92] Meelapsom R, Jarujamrus P, Amatatongchai M, et al. Talanta, 2016,155:193 27216673 10.1016/j.talanta.2016.04.037

[93] Chen G H, Chen W Y, Yen Y C, et al. Anal Chem, 2014,86(14):6843 24932699 10.1021/ac5008688

[94] Yan Z N, Jiang L, Xu C Y, et al. Water Sci Tech-W Sup, 2018,18(6):2081

[95] Guo J F, Huo D Q, Yang M, et al. Talanta, 2016,161:819 27769488 10.1016/j.talanta.2016.09.032

[96] Rattanarat P, Dungchai W, Cate D, et al. Anal Chem, 2014,86(7):3555 24576180 10.1021/ac5000224

[97] Shi J J, Tang F, Xing H L, et al. J Braz Chem Soc, 2012,23(6):1124

[98] Qi J, Li B W, Wang X R et al. Sensor Actuat B: Chem, 2017,251:224

[99] Zhou J R, Li B W, Qi A J, et al. Sensor Actuat B: Chem, 2020,305:127462

[100] Shang Q P, Zhang P, Li H J, et al. J Innov Opt Hea, 2019,12(6):1950016

[101] Mentele M M, Cunningham J, Koehler K, et al. Anal Chem, 2012,84(10):4474 22489881 10.1021/ac300309c

[102] Devadhasan J P, Kim J. Sensor Actuat B: Chem, 2018,273:18

[103] Statham P J. Sci Total Environ, 2012,434:213 22119025 10.1016/j.scitotenv.2011.09.088

[104] Kassir L N, Lartiges B, Ouaini N. Environ Technol, 2012,33(7/9):873 22720412 10.1080/09593330.2011.601765

[105] Molins-Legua C, Meseguer-Lloret S, Moliner-Martinez Y, et al. TrAC Trends in Anal Chem, 2006,25(3):282

[106] Jayawardane B M, McKelvie I D, Kolev S D. Anal Chem, 2015,87(9):4621 25855368 10.1021/acs.analchem.5b00125

[107] Peters J J, Almeida M, O'Connor Sraj L, , et al. Anal Chim Acta, 2019,1079:120 31387702 10.1016/j.aca.2019.05.050

[108] De Mey E, De Maere H, Paelinck H, et al. Crit Rev Food Sci Nutr, 2017,57(13):2909 26528731 10.1080/10408398.2015.1078769

[109] Lopez-Ruiz N, Curto V F, Erenas M M, et al. Anal Chem, 2014,86(19):9554 25158126 10.1021/ac5019205

[110] Trofimchuk E, Hu Y, Nilghaz A, et al. Food Chem, 2020,316:126396 32066068 10.1016/j.foodchem.2020.126396

[111] Nalin R, Wijitar D. J Anal Chem, 2020,75(4):487

[112] Razzaque M S. Clin Sci (Lond), 2011,120(3):91 20958267 10.1042/CS20100377 PMC3120105

[113] Racicot J M, Mako T L, Olivelli A, et al. Sensors (Basel), 2020,20(10):2766 10.3390/s20102766 PMC7294414 32408677

[114] Sarwar M, Leichner J, Naja G M, et al. Microsyst Nanoeng, 2019,5(1):56 31645999 10.1038/s41378-019-0096-8 PMC6803704

[115] Jayawardane B M, Wongwilai W, Grudpan K, et al. J Environ Qual, 2014,43(3):1081 25602837 10.2134/jeq2013.08.0336

[116] Gilbert-Lopez B, Garcia-Reyes J F, Molina-Diaz A. Talanta, 2009,79(2):109 19559852 10.1016/j.talanta.2009.04.022

[117] Xu Z, Fang G, Wang S. Food Chem, 2010,119(2):845

[118] Velkoska-Markovska L, Petanovska-Ilievska B. Acta Chromatographica, 2020,32(4):256

[119] Liu M, Hashi Y, Song Y, et al. J Chromatogr A, 2005,1097(1/2):183 16257002 10.1016/j.chroma.2005.10.022

[120] Zhu J J, Chen Q, Kutsanedzie F Y H, , et al. Anal Methods, 2017,9(43):6186

[121] Hossain S M Z, Luckham R E, Smith A M, et al. Anal Chem, 2009,81(13):5474 19492815 10.1021/ac900660p

[122] Tian F, Lyu J, Shi J Y, et al. Sensor Actuat B: Chem., 2016,225:312

[123] Burnham S, Hu J, Anany H, et al. Anal Bioanal Chem, 2014,406(23):5685 24969469 10.1007/s00216-014-7985-3

[124] Wang C H, Wu J J, Lee G B. Sensor Actuat B: Chem, 2019,284:395

[125] Jokerst J C, Adkins J A, Bisha B, et al. Anal Chem, 2012,84(6):2900 22320200 10.1021/ac203466y

[126] de Noordhout C M, Devleesschauwer B, Angulo F J, et al. Lancet Infect Dis, 2014,14(11):1073 25241232 10.1016/S1473-3099(14)70870-9 PMC4369580

[127] Liu H X, Zhan F F, Liu F, et al. Biosens Bioelectron, 2014,62:38 24973541 10.1016/j.bios.2014.06.020

[128] Bisha B, Adkins J A, Jokerst J C, et al. J Vis Exp, 2014,88:e51414 10.3791/51414 PMC4188167 24962090

[129] Chen J L. Microchim Acta, 2017,184(5):1335

[130] Landers T F, Cohen B, Wittum T E, et al. Public Health Reports, 2012,127(1):4 22298919 10.1177/003335491212700103 PMC3234384

[131] Ngumba E, Kosunen P, Gachanja A, et al. Anal Methods, 2016,8(37):6720

[132] Farouk F, Niessen W M A. SN Applied Sci, 2020,2(3):483

[133] Nilghaz A, Lu X. Anal Chim Acta, 2019,1046:163 30482295 10.1016/j.aca.2018.09.041

[134] Xing C R, Liu L Q, Song S S, et al. Biosens Bioelectron, 2015,66:445 25499659 10.1016/j.bios.2014.12.004

[135] Spalding R F, Fulton J W. J of Contaminant Hydrology, 1988,2(2):139

[136] Ivy M A, Gallagher L T, Ellington A D, et al. Chem Sci, 2012,3(6):1773

[137] Pramanik S, Hu Z, Zhang X, et al. Chem, 2013,19(47):15964 10.1002/chem.201301194 24123511

[138] Wang C, Huang H, Bunes B R, et al. Sci Rep, 2016,6:25015 27146290 10.1038/srep25015 PMC4857100

[139] Sener H, Anilanmert B, Cengiz S. Chem Papers, 2017,71(5):971

[140] Brensinger K, Rollman C, Copper C, et al. Forensic Sci Int, 2016,258:74 26666592 10.1016/j.forsciint.2015.11.007

[141] Mirasoli M, Buragina A, Dolci L S, et al. Anal Chim Acta, 2012,721:167 22405316 10.1016/j.aca.2012.01.036

[142] Moram S S B, Byram C, Shibu S N, et al. ACS Omega, 2018,3(7):8190 31458956 10.1021/acsomega.8b01318 PMC6644453

[143] Taudte R V, Beavis A, Wilson-Wilde L, et al. Lab Chip, 2013,13(21):4164 23959203 10.1039/c3lc50609f

[144] Tawfik S M, Sharipov M Kakhkhorov S, et al. Adv Sci, 2019,6(2):1801467 10.1002/advs.201801467 PMC6343090 30693188

[145] Salles M O, Meloni G N, de Araujo W R, et al. Anal Methods, 2014,6(7):2047

[146] Pesenti A, Taudte R V, McCord B, , et al. Anal Chem, 2014,86(10):4707 24766256 10.1021/ac403062y

[147] Moron-Lopez J, Nieto-Reyes L, Molina S, et al. Sci Total Environ, 2020,736:139672 32502787 10.1016/j.scitotenv.2020.139672

[148] Ding Y, Mutharasan R. Environ Sci Technol, 2011,45(4):1490 21189000 10.1021/es1020795

[149] Cao L H, Huang F Y, Massey I Y, et al. Toxins (Basel), 2019,11(9):482 10.3390/toxins11090482 PMC6783826 31438657

[150] Ge S G, Liu W Y, Ge L, et al. Biosens Bioelectron, 2013,49:111 23728196 10.1016/j.bios.2013.05.010

[151] Wang L B, Chen W, Xu D H, et al. Nano Letters, 2009,9:4147 19928776 10.1021/nl902368r PMC2793542

[152] Cuartero M, Crespo G A, Bakker E. Anal Chem, 2015,87(3):1981 25565062 10.1021/ac504400w

[153] Loh L J, Bandara G C, Weber G L, et al. Analyst, 2015,140(16):5501 26161586 10.1039/c5an00807g

[154] Lan W J, Zou X U, Hamedi M M, et al. Anal Chem, 2014,86(19):9548 25197763 10.1021/ac5018088

